# A homozygous missense variant in *HSD17B4* identified in a consanguineous Chinese Han family with type II Perrault syndrome

**DOI:** 10.1186/s12881-017-0453-0

**Published:** 2017-08-23

**Authors:** Kui Chen, Ke Yang, Su-Shan Luo, Chen Chen, Ying Wang, Yi-Xuan Wang, Da-Ke Li, Yu-Jie Yang, Yi-Lin Tang, Feng-Tao Liu, Jian Wang, Jian-Jun Wu, Yi-Min Sun

**Affiliations:** Department & Institute of Neurology, Huashan Hospital, Fudan University, 12 Wulumuqi Zhong Road, Shanghai, 200040 China

**Keywords:** Perrault syndrome, *HSD17B4*, Variant, Neurological features, Ovarian dysgenesis, Sensorineural deafness

## Abstract

**Background:**

Perrault syndrome is a rare multisystem disorder that manifests with sensorineural hearing loss in both sexes, primary ovarian insufficiency in females and neurological features. The syndrome is heterogeneous both genetically and phenotypically.

**Case presentation:**

We reported a consanguineous family (two affected sisters) with Perrault syndrome. The proband had the characteristics of Perrault syndrome: ovarian dysgenesis, bilateral hearing loss and obvious neurological signs. Target genetic sequencing and triplet repeat primed PCR (TP-PCR) plus capillary electrophoresis was conducted to detect causative mutations in the proband. The detected variant was further confirmed in the proband and tested in other family members by Sanger sequencing. Both the proband and her sister were found homozygous for the novel variant *HSD17B4* c.298G > T (p.A100S) with their parents heterozygous. Detected by western blot, the protein expression of *HSD17B4* mutant was much lower than that of the wild type in SH-SY5Y cells transfected by *HSD17B4* wild type or mutant plasmid, which indicated the pathogenicity of the *HSD17B4* mutation.

**Conclusions:**

Our findings supported that *HSD17B4* was one of the genes contributing to Perrault syndrome with the likely pathogenic variant c.298G > T (p.A100S). Special manifestations of cerebellar impairment were found in cases caused by *HSD17B4* mutations. Besides, attention should be paid to distinguish Perrault syndrome from D-bifunctional protein deficiency and hereditary ataxia.

**Electronic supplementary material:**

The online version of this article (doi:10.1186/s12881-017-0453-0) contains supplementary material, which is available to authorized users.

## Background

Perrault syndrome (PRLTS, MIM233400), a rare autosomal-recessive disorder influenced by sex, is characterized by sensorineural hearing loss in both male and female patients and ovarian dysgenesis in female patients [1]. A wide variety of additional clinical features including peripheral neuropathy, marfanoid habitus, cerebellar ataxia, intellectual disability and spasticity, most neurologically, have also been reported [2–4]. The clinical heterogeneity has prompted a suggested classification of PRLTS into type I and type II, without and with neurological symptoms respectively [2, 5]. However, the determinants for different types remain unknown.

With further evidence, it is likely that the genetic heterogeneity has relationship with the phenotypic heterogeneity. Mutations in four genes including *HSD17B4* (MIM601860), *HARS2* (MIM600783), *CLPP* (MIM601119) and *LARS2* (MIM604544) have been identified as genetic causes of PRLTS [5–9]. However, the association between genotype and phenotype remains uncertain.

*HSD17B4* locates on chromosome 5q23.1, encoding an enzyme of 17β-hydroxysteroid dehydrogenase type 4 [10]. This multifunctional peroxisomal enzyme, also known as D-bifunctional protein (DBP), acts an important role in fatty acid β-oxidation metabolism [11]. Therefore, besides PRLTS, *HSD17B4* is also related to DBP deficiency (DBPD, MIM261515). DBPD is an autosomal-recessive and infantile-onset disorder characterized by severe neurological symptoms and accumulation of very long chain fatty acid (VLCFA) in plasma [11, 12]. Compared with DBPD patients, PRLTS patients present with both of hearing loss and gonadal dysgenesis, while their VLCFA level is normal.

Since PRLTS is a rare disease, there are only approximately 40 PRLTS families reported worldwide [13], among which three cases were possibly caused by mutations in *HSD17B4* [5, 9, 14, 15].

Here we gave the report of a PRLTS family in China and found an *HSD17B4* mutation to confirm the relationship. Furthermore, we compared PRLTS and DBPD to clarify the diagnosis. In the end, we summarized the PRLTS cases with *HSD17B4* mutations.

## Case presentation

### Subjects and clinical evaluation

**Fig. 1:**
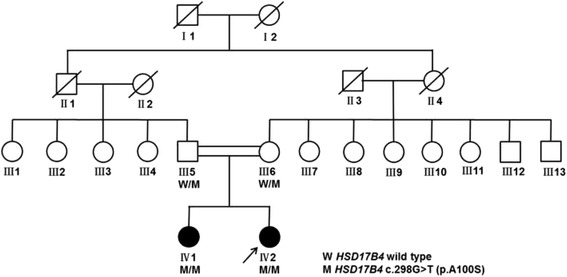
Pedigree of the consanguineous Perrault syndrome family. Affected individuals were denoted by filled symbols. The arrow indicated the proband (IV2). Genotypes were shown below each symbol

We collected a PRLTS family with two affected siblings (Fig. 1) from eastern China. The proband (IV2) was subjected to laboratory tests, audiometric examination, thorough neurological examinations, pelvic ultrasonography (iU22 ultrasonography unit, Philips Medical Systems, Bothell, WA) and cranial magnetic resonance imaging (MRI) (3 T MRI scanner Siemens, Erlangen, Germany). The other family members were evaluated by interviews. The CARE guidelines were followed in this case.

### Genetic testing and variants screening

Genomic DNA samples were extracted from peripheral blood leucocytes of all family members. For the proband (IV2), a panel containing 218 genes of hereditary spastic paraplegia and ataxia was performed by target sequencing of the exons. Gene list of the panel was in Additional file 1: Table S1. High throughput sequencing was carried out via the HiSeq2500 sequencer (Illumina, San Diego, CA). All variants different from the reference sequence were further screened by allele frequency < 1% according to 1000 Genomes Project (http://www.internationalgenome.org/data), Inhouse database and ESP6500 (evs.gs.washington.edu/EVS/). The synonymous variants were excluded. The phenotypes of the screened genes were compared with the clinical manifestations of the proband and the inherited modes were considered to further exclude irrelevant genes. Then the mutations left were tested in other members by Sanger sequencing for family segregation.

The dynamic mutations of Spinocerebellar ataxia (SCA) type 1, 2, 3, 6, 7, 12, 17 and Dentatorubral-pallidoluysian atrophy (DRPLA) were detected by triplet repeat primed PCR (TP-PCR) and capillary electrophoresis.

SIFT (http://sift.jcvi.org/), Polyphen-2 (http://genetics.bwh.harvard.edu/pph2/), and MutationTaster (http://www.mutationtaster.org/) computer simulation software programs were used to predict the potential of the variants to affect function.

### Construction of recombinant plasmids

PCR fragments encoding the mutant or wild-type *HSD17B4* gene were amplified using a pair of designed primers (forward nucleotide sequence: CGCAAATGGGCGGTAGGCGTG and reverse nucleotide sequence: TAGAAGGCACAGTCGAGG). The cDNA was then cloned into the pcDNA3.1–3xFlag vector via the Xhol and BamHI restriction sites. Both constructs were confirmed by sequencing.

### Cell culture and transfection

SH-SY5Y cells were used throughout this study. The cells were cultured in DMEM media supplemented with 10% fetal bovine serum and plated into wells of a six-well plate with a density of 3×10^5^ cells/ml. Transfections were carried out using Lipofectamine 2000 from Life Technologies (Invitrogen) according to the manufacturer’s protocol. The experiments were repeated separatedly for three times.

### Western blotting

Following transfection for 36 h, total proteins were extracted with cell lysis buffer for western blotting (Beyotime) containing of Protease Inhibitor Cocktail (Thermo). Equal amounts of protein were diluted into 5× loading buffer to 20 ml. After electrophoresis, the separated proteins were transferred to Nitrocellulose membranes. Then the membranes were blocked in TBST containing 5% non-fat milk for 1 h and incubated overnight with primary antibodies, rabbit anti-*HSD17B4* (Dilution 1:100, HPA021479, Sigma) and mouse anti-beta-actin (Dilution 1:1000, #M20011, Abmart). Next, the membranes were incubated for 1 h with a matching secondary antibody at room temperature. Protein expression was detected using the Immobilon Western Chemiluminescent HRP Substrate. After normalization to actin, relative protein expression was calculated by Imagelab.

### Clinical features

**Fig. 2:**
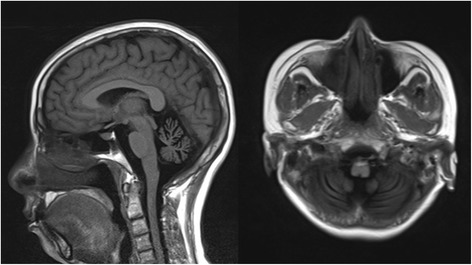
Brain MRI images for the proband (IV2). Marked and diffuse cerebellar atrophy was present in the proband

This family was consanguineous and consisted of the female proband (37-year old), her affected sister (40-year old) and their unaffected relatives (Fig. 1). The proband referred to the neurology clinic initially for ataxia described as the deteriorated ability of balance leading to occasional falls. She also had dysarthria for seven years. In the nervous system examination, she presented with ataxic gait, mild hypertonia in lower limbs, hyperactive deep tendon reflexes, while muscle strength and sensory conduction were normal, and there was no obvious dysmorphology. She performed badly in finger-nose test, alternating movement test and straight-line walking. In addition, she had nystagmus on lateral gaze and hypermetric saccades, but there was no limitation in extraocular movements. We could also find modest dysarthria and mildly intellectual impairment. Her Mini-Mental State Examination score was 25 and considered as mild cognitive impairment according to her education year of 9 [16]. The cranial MRI showed moderate atrophy of the cerebellum (Fig. 2).

The patient also had secondary amenorrhea which needed the endocrine therapy to keep the regular menstrual cycle. But she had stopped the therapy a few years ago. According to her hormone profile, luteinizing hormone (LH) and follicle stimulating hormone (FSH) were elevated, and estradiol was low (Table 1). Furthermore, the uterus appeared small (40×25×20mm) with endometrial stripes and indistinct ovaries on ultrasound. These results were consistent with hypergonadotropic hypogonadism.

**Table 1:** Molecular and clinical details for PRLTS patients with *HSD17B4* mutations

Family [Reference]	1 [5,15]			2 [9]		3 [14]		4 [This case]	
Affected member	P1–1		P1–2	P2		P3		P4–1	P4–2
Variant	c.650A > G;p.Y217C	c.1704 T > A;p.Y568X	The same as P1–1	c.46G > A;p.G16S	c.244G > T;p.V82F	c.317G > A;p.R106H	c.1675A > G;p.I559V	c.298G > T;p.A100S	The same as P4–1
Ref Seq transcript	NM_000414			NM_000414		NM_000414		NM_000414	
HET/HOM	Compound HET	Compound HET		Compound HET	Compound HET	HOM	HOM	HOM	
Mutation type	Missense	Nonsense		Missense	Missense	Missense	Missense	Missense	
Affected domain	DD	HD		DD	DD	DD	HD	DD	
Ethnicity	American of mixed European ancestry			Brazilian		Korean		Chinese	
Consanguinity	N			N		N		Y	
Sex	F		F	F		F		F	F
Age at last assessment, years	27		16	43		15		37	40
Gonadal dysgenesis									
Amenorrhea	Primary		NA	NR		Primary		Secondary	Primary
Pelvic US/MRI	NR		NR	small uterus and ovaries		hypoplastic and vagina, and ovarian agenesis.		small uterus and ovaries	NR
FSH(RI),IU/L	111		NR	72 (0.9–15)		108.76 (0.3–9.0)		59.80 (3.50–12.50)	NR
LH(RI),IU/L	81.89		NR	59 (1.3–13)		21.8 (0.1–10.6)		30.08 (2.40–12.60)	NR
Estradiol(RI),pg/mL	NR		Low	12.6		12.01 (20–50)		7.6 (12.5–16.3)	NR
Testosterone(RI),ng/dL	NA		NA	NA		NA		NA	NA
Azoospermia	NA		NA	NA		NA		NA	NA
SNHL	++/+++		++/+++	+++		+++left ear		++	+
Neurological features									
Motor									
Weakness	+,LE		+	NR		NR		−	−
LE spasticity	+		NR	NR		NR		+	−
Deep tendon reflexes	NR		NR	NR		NR		++	−
Pes cavus	Y		NR	NR		NR		−	−
Sensory conduction	Impaired		NR	NR		NR		−	−
Cerebellum									
Ataxia	Y		−	Y				Y	Y
Mobility aids	Wheelchair		−	NR		−		−	−
Intension tremor	Y		Y	NR		NR		−	−
Nystagmus	Y(on lateral gaze)		−	NR		NR		Y(on lateral gaze)	NR
Oculomotor apraxia	Y		−	NR		NR		Y	−
Dysarthria			+	NR		NR		Y	Y
Cranial MRI									
Cerebellar atrophy	++/+++		NR	Y		NR		++	NR
Intellecture disability	NR		NR	+		NR		+	+
Growth retardation	+		−	NR		+		−	−

*Abbreviations*: *HET* heterozygous, *HOM* homozygous, *DD* dehydrogenase domain, *HD* hydratase domain, *N* no, *Y* yes, *F* female, *M* male, *NR* not recorded, *NA* not applicable, *LE* lower extremities, *SNHL* sensorineural hearing loss, *FSH* follicle-stimulating hormone, *LH* luteinizing hormone, *RI* reference interval, *NCV* nerve conduction velocities, *VLCFA* very long chain fatty acids; +, mild; ++, moderate; +++, severe; −, normal

**Fig. 3:**
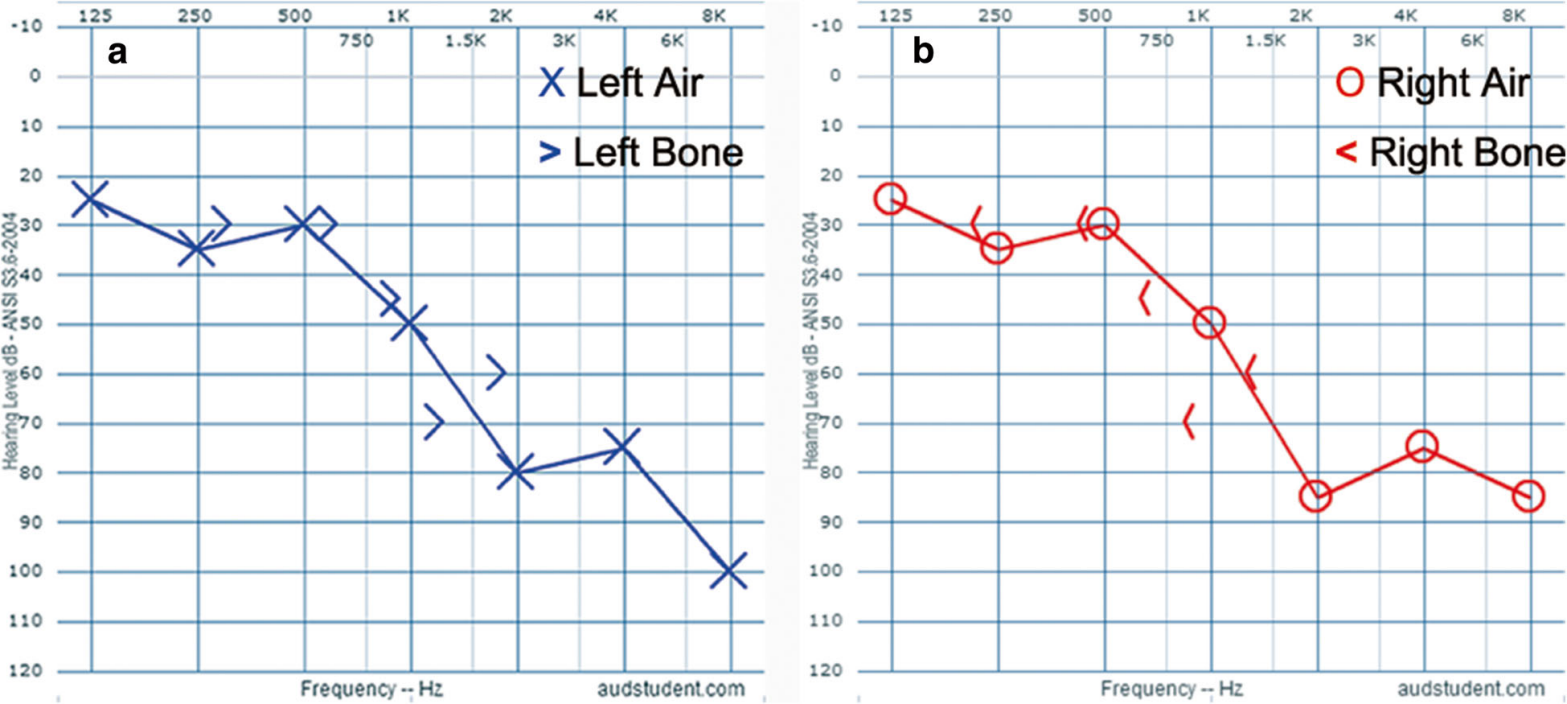
Audiograms from the proband at the age of 37. **a** Audiogram of the left ear. **b** Audiogram of the right ear. In pure-tone audiometry measurements, similar air and bone thresholds are indicative of hearing loss due to dysfunction of the inner ear, which demonstrates bilaterally sensorineural hearing loss especially at high frequency. Audiograms were created using the AudGen online tool (version 0.71) (http://audsim.com/audgen/)

Another clinical feature was hearing impairment. The proband had hearing dysfunction from childhood without great progression in these years. Audiometric examination showed a bilaterally sensorineural hearing deficit for higher frequencies (Fig. 3).

In addition, her VLCFA was normal (Table 2).

**Table 2:** VLCFA concentration of the proband of the Perrault syndrome pedigree

VLCFA	Units	The proband (IV2)	Reference range
C22:0	nmol/ml	47.6	≤96.3
C24:0	nmol/ml	22.1	≤91.4
C26:0	nmol/ml	<0.40	≤1.30
C24:C22		0.47	≤1.39
C26:C22		<0.008	≤0.023

*Abbreviations*: *VLCFA* very long chain fatty acids

The proband’s sister (IV1) had similar symptoms as the proband. She had primary amenorrhea and tried several therapies all of which ended with failure. She also suffered from the hearing loss. And she had the tendency to have a fall in daily life. She was unwilling to take the hormone test, ultrasound test of uterus, VLCFA or cerebral MRI scan. Both of their parents were healthy.

### Genetic information

The mean depth of target sequencing of the patient was 1083.5× and the coverage was 100%. The percentage of the target region with mean depth > 20× was 100%. 29 variants left after screening by the variants frequencies and synonymous status (Additional file 2: Table S2). According to the autosomal recessive inheritance mode in this family, we complemented the variants by homozygosity mapping. Two variants of c.298G > T (p.A100S) (accession no. NM_001199292) in *HSD17B4* and c.188A > G (p.D63G) in *KCNC3* (NM_004977) were found with a sequencing depth of 663× and 66× respectively. The variant in *KCNC3* was excluded since it was associated with spinocerebellar ataxia 13 whose manifestations were different from this patient. This variant of p.A100S in *HSD17B4* was not found in 1000 Genomes Project, Inhouse or ESP6500 database. The predicted values of this variant were 0, 1 and 1 in SIFT, PolyPhen and MutationTaster respectively, which resulted in the conclusion of a damaging variant in silico algorithms. Sanger sequencing of other genes associated with PRLTS, including *HARS2*, *CLPP* and *LARS2,* was conducted with negative results. Primer information could be found in Additional file 3: Table S3.

**Fig. 4:**
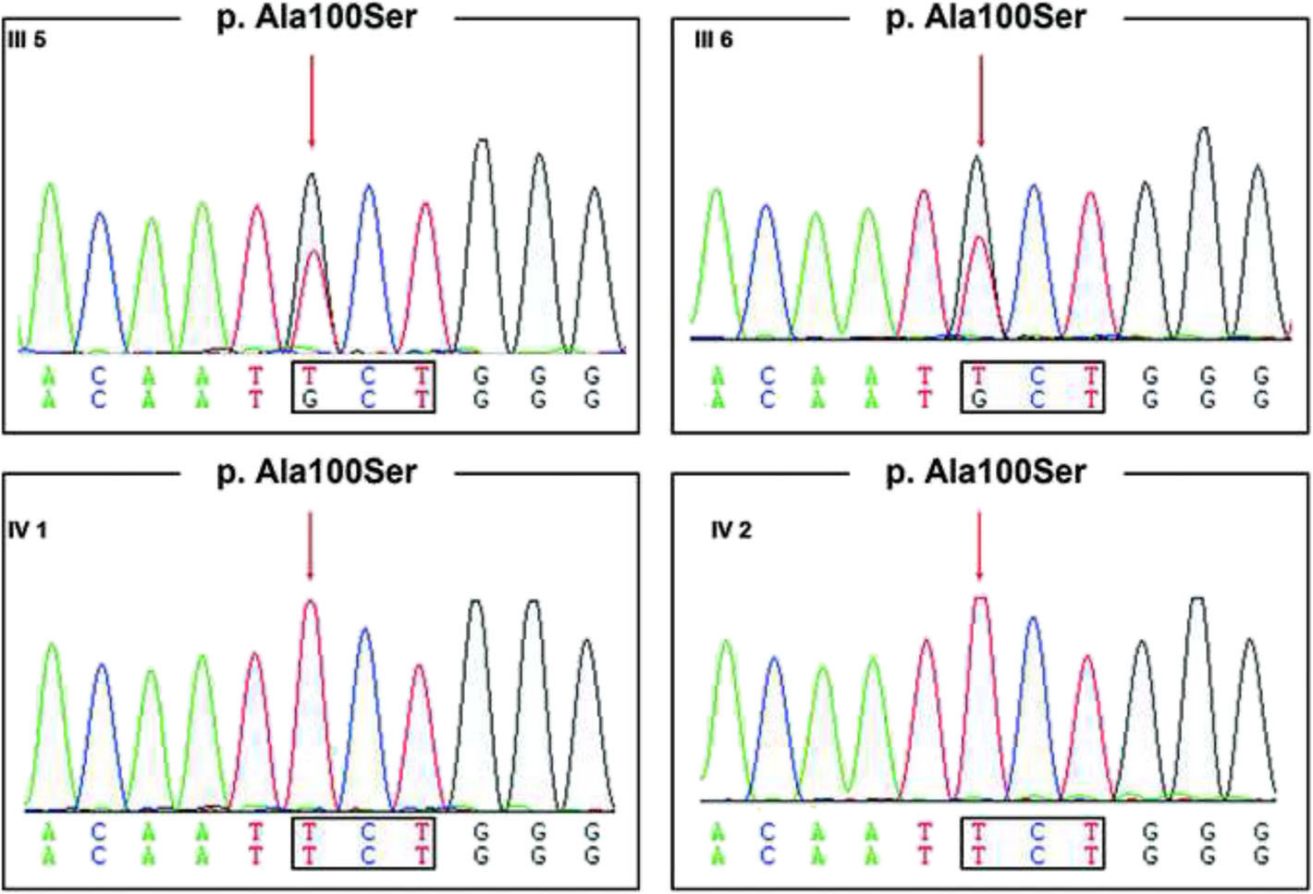
Sanger sequences of the mutation in *HSD17B4* in the affected sisters and their parents. The position of the G > T transition, which led to alanine to serine substitution, was indicated by an arrow. The mutation could be found in heterozygous parents (III5 and III6) and affected patients (IV1 and IV2)

The variant was then confirmed in the proband and investigated in other family members by Sanger sequencing. Her sister was also found homozygous, with both parents heterozygotes (Figs. 1 and 4).

The dynamic mutations in SCA1–3, 6, 7, 12, 17 and DRPLA were not found.

### Expression of the mutant gene

**Fig. 5:**
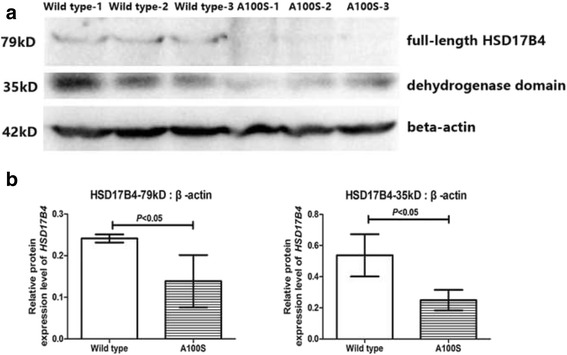
Analysis of wild-type and mutant HSD17B4 protein expression by Western blot. **a** Compared by T-test, expression of the variant c.298G > T (p.A100S) was lower than expression of the wild-type. **b** There was significant difference between protein expressions for transfected wild-type and mutant plasmids

Western blot analysis revealed that the 79kD full-length DBP protein and the 35 kD dehydrogenase domain were detected in cells transfected with a wild-type construct. However, there was remarkable reduction in protein expression resulted from the variants c.298G > T (p.A100S) in *HSD17B4*. There was significant difference between the protein expression levels of the wild type and the variant (Fig. 5).

## Discussion and conclusions

The proband in this family had clinical hallmarks of Perrault syndrome. Given that she had gonadal dysgenesis, hearing loss and neurological disorders, she could be categorized as type II Perrault Syndrome. In this family, we identified a novel missense variant *HSD17B4* p.A100S. According to the guidelines of interpretation of sequence variants of the American College of Medical Genetics and Genomics (ACMG), this mutation was classified as a likely pathogenic variant [17]. The patient’s familial history and clinical characteristics were specific. And variants in other suspected genes were not found by the target sequencing. Moreover, mutations in *HSD17B4* would result in the alteration in the dehydrogenase domain of DBP and had been identified as the possible cause for Perrault syndrome in several families [14, 18]. In this case we have demonstrated that *HSD17B4* p.A100S had  a damaging effect on the gene product by western blot. On the basis of these evidences, this homozygous variant was considered as the probable cause of the disease.

As mentioned above, *HSD17B4* encodes the protein peroxisomal enzyme DBP that catalyzes the β-oxidation of VLCFA. There are three domains in DBP: a 3-hydroxyacyl-CoA dehydrogenase domain, a 2-enoyl-CoA hydratase domain, and a sterol carrier protein 2-like domain [19]. After peroxisomal import, a hydratase unit is obtained from the proteolytically cleaving of DBP, with a dehydrogenase unit produced meanwhile [20, 21]. These two domains catalyze the hydration and dehydrogenation steps in β-oxidation of the VLCFA sequentially [22]. The mutant p.A100 residue in this case was located at the dehydrogenase region of DBP [5]. *HSD17B4* p.A100S was predicted by several algorithms to be damaging.

*HSD17B4* variants can lead to PRLTS as well as DBPD. These two diseases also have some similar manifestations so that it is necessary to differentiate PRLTS from DBPD. DBP dysfunction could result in the accumulation of β-oxidation substrates such as VLCFA, which is one of the most significant characteristics of DBPD typesI, II and III. In most cases of DBPD typesI, II and III, the patients presented with severe manifestations including hypotonia and seizures by one month of age and die within the first two years of life [12]. These patients also had dysmorphic features, visual and hearing impairment, and growth retardation [11]. According to the deficient activity, DBPD can be divided into three subtypes [23]. Type I is the result of deficiencies of both the hydratase and dehydrogenase subunits of DBP, while types II and III have isolated deficiency of hydratase or dehydrogenase. However, a novel type of DBPD has been proposed recently [24]. Patients with the juvenile-onset type, also known as type IV, show mildly clinical features of hearing loss, ataxia, retinal atrophy and fertility in males and females with compound heterozygous mutations affecting both subunits in separate alleles. The juvenile-onset DBPD seems to have more various symptoms and higher VLCFA level than PRLTS. However, the VLCFA in some DBPD patients could be normal as well [2, 25]. On account of the similar cause and manifestation, PRLTS due to *HSD17B4* mutations and juvenile-onset DBP deficiency may be the same disease or part of the same disease spectrum. In our case, besides milder course of the disease, hearing loss and neurological signs, the proband had moderate ovarian dysgenesis, an essential characteristic of PRLTS. In addition, her VLCFA level was normal, and there was only cerebellar atrophy in MRI which was different from the “peroxisomal pattern” [26]. The “peroxisomal pattern” was considered as a diagnostic clue for DBPD when the VLCFA level was normal. Typically, there was cerebral and cerebellar leukencephalopathy, perisylvic polymicrogyria, and frontoparietal pachygyria in the “peroxisomal pattern”. While in the cases of PRLTS, there was usually cerebellar atrophy. These features made us tend to the diagnosis of PRLTS rather than the juvenile-onset DBPD. Nevertheless it remained to investigate the relationship between these two diseases associated with *HSD17B4*.

Based on this case, we summarized definite or probable PRLTS cases involving *HSD17B4* mutations (Table 1) [5, 9, 14, 15]. Interestingly, these patients presented with various neurological symptoms, including signs of cerebellar dysfunction, ocular anomalies and intellectual disability. Among these, cerebellar manifestations were obvious, which could also be demonstrated by the cerebellar atrophy in MRI. Compared with that, the symptoms relevant to peripheral nervous system seemed to be milder. Hearing loss and gonadal dysgenesis, the typical features of PRLTS, were developed in all patients with *HSD17B4* mutations. In other PRLTS cases without *HSD17B4* variants, either marfanoid features or short stature was reported [27, 28], both of which were not shown characteristically in the patients with *HSD17B4* mutations except for pes cavusin. In our case, ataxia was the chief complaint and this might lead to misdiagnosis if the symptoms of ovarian dysgenesis and hearing loss were ignored.

This report genetically confirmed a PRLTS family in China. This family was consanguineous and the patients were homozygous for *HSD17B4* c.298G > T (p.A100S). In review of PRLTS patients with *HSD17B4* mutation, we found that these patients presented with various neurological symptoms. More cases could be collected to find whether there was an association between *HSD17B4* mutations and PRLTS phenotype. Besides, the mechanism remains to investigate that how the mutations in *HSD17B4* lead to PRLTS and DBPD.

## Additional files

Additional file 1: Table S1. Gene list in the panel performed by target sequencing of the exons. (DOCX 29 kb)

Additional file 2: Table S2. List of Variants in the proband. 29 variants were left after screening by the variants frequencies and synonymous status. (XLSX 26 kb)

Additional file 3: Table S3. Primer information of *HARS2*, *CLPP* and *LARS2* for Sanger sequencing. (XLS 27 kb)

## Abbrevations

ACMG: American College of Medical Genetics and Genomics
DBP: D-bifunctional protein
DBPD: DBP deficiency
DRPLA: Dentatorubral-pallidoluysian atrophy
FSH: Follicle stimulating hormone
LH: Luteinizing hormone
OMIM: Online Mendelian Inheritance in Man
PRLTS: Perrault syndrome
SCA: Spinocerebellar ataxia
VLCFA: Very long chain fatty acid
VUS: Variant of uncertain significance

## References

[1] Perrault M, Klotz B, Housset E (1951). Two cases of turner syndrome with deaf-mutism in two sisters. Bull Mem Soc Med Hop Paris.

[2] Fiumara A, Sorge G, Toscano A, Parano E, Pavone L, Opitz JM (2004). Perrault syndrome: evidence for progressive nervous system involvement. Am J Med Genet A.

[3] Linssen WH, Van den Bent MJ, Brunner HG, Poels PJ (1994). Deafness, sensory neuropathy, and ovarian dysgenesis: a new syndrome or a broader spectrum of Perrault syndrome?. Am J Med Genet A.

[4] Gottschalk ME, Coker SB, Fox LA (1996). Neurologic anomalies of Perrault syndrome. Am J Med Genet.

[5] Pierce SB, Walsh T, Chisholm KM, Lee MK, Thornton AM, Fiumara A, Opitz JM, Levy-Lahad E, Klevit RE, King MC (2010). Mutations in the DBP-deficiency protein HSD17B4 cause ovarian dysgenesis, hearing loss, and ataxia of Perrault syndrome. Am J Med Genet.

[6] Pierce SB, Chisholm KM, Lynch ED, Lee MK, Walsh T, Opitz JM, Li W, Klevit RE, King MC (2011). Mutations in mitochondrial histidyl tRNA synthetase HARS2 cause ovarian dysgenesis and sensorineural hearing loss of Perrault syndrome. Proc Natl Acad Sci U S A.

[7] Pierce SB, Gersak K, Michaelson-Cohen R, Walsh T, Lee MK, Malach D, Klevit RE, King MC, Levy-Lahad E (2013). Mutations in LARS2, encoding mitochondrial leucyl-tRNA synthetase, lead to premature ovarian failure and hearing loss in Perrault syndrome. Am J Med Genet.

[8] Jenkinson EM, Rehman AU, Walsh T, Clayton-Smith J, Lee K, Morell RJ, Drummond MC, Khan SN, Naeem MA, Rauf B (2013). Perrault syndrome is caused by recessive mutations in CLPP, encoding a mitochondrial ATP-dependent chambered protease. Am J Med Genet.

[9] Demain LA, Urquhart JE, O'Sullivan J, Williams SG, Bhaskar SS, Jenkinson EM, Lourenco CM, Heiberg A, Pearce SH, Shalev SA (2017). Expanding the Genotypic Spectrum of Perrault syndrome. Clin Genet.

[10] Lines MA, Jobling R, Brady L, Marshall CR, Scherer SW, Rodriguez AR, Lee L, Lang AE, Mestre TA, Wanders RJ (2014). Peroxisomal D-bifunctional protein deficiency: three adults diagnosed by whole-exome sequencing. Neurology.

[11] de Launoit Y, Adamski J (1999). Unique multifunctional HSD17B4 gene product: 17beta-hydroxysteroid dehydrogenase 4 and D-3-hydroxyacyl-coenzyme a dehydrogenase/hydratase involved in Zellweger syndrome. J Mol Endocrinol.

[12] Ferdinandusse S, Denis S, Mooyer PA, Dekker C, Duran M, Soorani-Lunsing RJ, Boltshauser E, Macaya A, Gartner J, Majoie CB (2006). Clinical and biochemical spectrum of D-bifunctional protein deficiency. Ann Neurol.

[13] Sampathkumar G, Veerasigamani N (2015). Perrault syndrome - a rare case report. J Clin Diagn Res.

[14] Kim MJ, Kim SJ, Kim J, Chae H, Kim M, Kim Y (2013). Genotype and phenotype heterogeneity in perrault syndrome. J Pediatr Adolesc Gynecol.

[15] McCarthy DJ, Opitz JM (1985). Perrault syndrome in sisters. Am J Med Genet.

[16] Guo QH, Cao XY, Zhou Y, Zhao QH, Ding D, Hong Z (2010). Application study of quick cognitive screening test in identifying mild cognitive impairment. Neurosci Bull.

[17] Richards S, Aziz N, Bale S, Bick D, Das S, Gastier-Foster J, Grody WW, Hegde M, Lyon E, Spector E (2015). Standards and guidelines for the interpretation of sequence variants: a joint consensus recommendation of the American College of Medical Genetics and Genomics and the Association for Molecular Pathology. Genet Med.

[18] Lieber DS, Hershman SG, Slate NG, Calvo SE, Sims KB, Schmahmann JD, Mootha VK (2014). Next generation sequencing with copy number variant detection expands the phenotypic spectrum of HSD17B4-deficiency. BMC Med Genet.

[19] Leenders F, Husen B, Thole HH, Adamski J (1994). The sequence of porcine 80 kDa 17 beta-estradiol dehydrogenase reveals similarities to the short chain alcohol dehydrogenase family, to actin binding motifs and to sterol carrier protein 2. Mol Cell Endocrinol.

[20] Jiang LL, Miyazawa S, Souri M, Hashimoto T (1997). Structure of D-3-hydroxyacyl-CoA dehydratase/D-3-hydroxyacyl-CoA dehydrogenase bifunctional protein. J Biochem.

[21] van Grunsven EG, van Berkel E, Mooijer PA, Watkins PA, Moser HW, Suzuki Y, Jiang LL, Hashimoto T, Hoefler G, Adamski J (1999). Peroxisomal bifunctional protein deficiency revisited: resolution of its true enzymatic and molecular basis. Am J Med Genet.

[22] Baes M, Huyghe S, Carmeliet P, Declercq PE, Collen D, Mannaerts GP, Van Veldhoven PP (2000). Inactivation of the peroxisomal multifunctional protein-2 in mice impedes the degradation of not only 2-methyl-branched fatty acids and bile acid intermediates but also of very long chain fatty acids. J Biol Chem.

[23] Moller G, van Grunsven EG, Wanders RJ, Adamski J (2001). Molecular basis of D-bifunctional protein deficiency. Mol Cell Endocrinol.

[24] McMillan HJ, Worthylake T, Schwartzentruber J, Gottlieb CC, Lawrence SE, Mackenzie A, Beaulieu CL, Mooyer PA, Wanders RJ, Majewski J (2012). Specific combination of compound heterozygous mutations in 17beta-hydroxysteroid dehydrogenase type 4 (HSD17B4) defines a new subtype of D-bifunctional protein deficiency. Orphanet J Rare Dis.

[25] Amor DJ, Marsh AP, Storey E, Tankard R, Gillies G, Delatycki MB, Pope K, Bromhead C, Leventer RJ, Bahlo M (2016). Heterozygous mutations in HSD17B4 cause juvenile peroxisomal D-bifunctional protein deficiency. Neurology Genetics.

[26] Gronborg S, Kratzner R, Spiegler J, Ferdinandusse S, Wanders RJ, Waterham HR, Gartner J (2010). Typical cMRI pattern as diagnostic clue for D-bifunctional protein deficiency without apparent biochemical abnormalities in plasma. Am J Med Genet A.

[27] Nikolaou DS, Winston RM (1999). Sporadic Perrault syndrome. J Obstet Gynaecol.

[28] Nishi YHK, Kajiyama M, Kawamura I (1988). The Perrault syndrome: clinical report and review. Am J Med Genet.

