# Supplementary material for: A homozygous missense variant in HSD17B4 identified in a consanguineous Chinese Han family with type II Perrault syndrome
Source: BMC Med Genet. 2017 Aug 23;18:91. doi: 10.1186/s12881-017-0453-0 (PMC5568266; doi:10.1186/s12881-017-0453-0)
Supplement: Supplementary file 1 — Gene list in the panel performed by target sequencing of the exons. (DOCX 29 kb) [file 12881_2017_453_MOESM1_ESM.docx]

**Additional file 1: Table S1. Gene list in the panel performed by target sequencing of the exons.**

| Symptom | Gene | Inheritane patterns | Symptom | Gene | Inheritance patterns |
| --- | --- | --- | --- | --- | --- |
| SCA1 | Ataxin-1 | AD | SCAR1 | SETX | AR |
| SCA2 | Ataxin-2 | AD | SCAR7 | TPP1 | AR |
| SCA3 | Ataxin-3 | AD | SCAR8 | SYNE1 | AR |
| SCA5 | SPTBN2 | AD | SCAR9 | CABC1 | AR |
| SCA6 | CACNA1A | AD | SCAR10 | ANO10 | AR |
| SCA7 | Ataxin-7 | AD | SCAR11 | SYT14 | AR |
| SCA8 | Ataxin-8 | AD | SCAR12 | WWOX | AR |
| SCA11 | TTBK2 | AD | SCAR13 | GRM1 | AR |
| SCA12 | PPP2R2B | AD | SCAR14 | SPTBN2 | AR |
| SCA13 | KCNC3 | AD | SCAR15 | KIAA0226 | AR |
| SCA14 | PRKCG | AD | SCAR16 | STUB1 | AR |
| SCA15/16 | ITPR1 | AD | SPG1 | L1CAM | XR |
| SCA17 | TBP | AD | SPG2 | PLP1 | XR |
| SCA19/22 | KCND3 | AD | SPG3 | ATL1 | AD |
| SCA23 | PDYN | AD | SPG4 | SPAST | AD |
| SCA26 | EEF2 | AD | SPG5 | CYP7B1 | AR |
| SCA27 | FGF14 | AD | SPG6 | NIPA1 | AD |
| SCA28 | AFG3L2 | AD | SPG7 | SPG7 | AR |
| SCA34 | ELOVL4 | AD | SPG8 | KIAA0196 | AD |
| SCA35 | TGM6 | AD | SPG9 | unkonwn | AD |
| SCA36 | NOP56 | AD | SPG10 | KIF5A | AD |
| DPRLA | ATN1 | AD | SPG11 | SPG11 | AR |
| ATXIA Syndrome | ATP1A3 | AD | SPG12 | RTN2 | AD |
| ATXIA Syndrome | CAMTA1 | AD | SPG13 | HSPD1 | AD |
| ATXIA Syndrome | DNMT1 | AD | SPG15 | ZFYVE26 | AR |
| ATXIA Syndrome | GFAP | AD | SPG17 | BSCL2 | AD |
| ATXIA Syndrome | IFRD1 | AD | SPG18 | ERLIN2 | AR |
| ATXIA Syndrome | ITM2B | AD | SPG20 | SPG20 | AR |
| ATXIA Syndrome | NOL3 | AD | SPG21 | SPG21 | AR |
| ATXIA Syndrome | PAX6 | AD | SPG22 | SLC16A2 | XR/XD |
| ATXIA Syndrome | POLG | AD | SPG26 | B4GALNT1 | AR |
| ATXIA Syndrome | PTEN | AD | SPG28 | DDHD1 | AR |
| ATXIA Syndrome | SCN1A | AD | SPG30 | KIF1A | AR |
| ATXIA Syndrome | SLC2A1 | AD | SPG31 | REEP1 | AD |
| ATXIA Syndrome | TUBB4A | AD | SPG33 | ZFYVE27 | AD |

| Symptom | Gene | Inheritane patterns | Symptom | Gene | Inheritance patterns |
| --- | --- | --- | --- | --- | --- |

| ATXIA Syndrome | VHL | AD | SPG35 | FA2H | AR |
| --- | --- | --- | --- | --- | --- |
| ATXIA Syndrome | COL18A1 | AR | SPG39 | PNPLA6 | AR |
| ATXIA Syndrome | TDP2 | AR | SPG42 | SLC33A1 | AD |
| ATXIA Syndrome | SNX14 | AR | SPG43 | C19orf12 | AR |
| ATXIA Syndrome | GOSR2 | AR | SPG44 | GJC2 | AR |
| ATXIA Syndrome | PRICKLE1 | AR | SPG46 | GBA2 | AR |
| ATXIA Syndrome | SCARB2 | AR | SPG47 | AP4B1 | AR |
| ATXIA Syndrome | KCTD7 | AR | SPG48 | AP5Z1 | AR |
| ATXIA Syndrome | PEX10 | AR | SPG49 | TECPR2 | AR |
| ATXIA Syndrome | TDP1 | AR | SPG50 | AP4M1 | AR |
| ATXIA Syndrome | PIK3R5 | AR | SPG51 | AP4E1 | AR |
| ATXIA Syndrome | GRID2 | AR | SPG52 | AP4S1 | AR |
| ATXIA Syndrome | EXOSC3 | AR | SPG53 | VPS37A | AR |
| ATXIA Syndrome | ATM | AR | SPG54 | DDHD2 | AR |
| ATXIA Syndrome | DNAJC19 | AR | SPG55 | C12orf65 | AR |
| ATXIA Syndrome | ATCAY | AR | SPG56 | CYP2U1 | AR |
| ATXIA Syndrome | SACS | AR | SPG61 | ARL6IP1 | AR |
| ATXIA Syndrome | ERCC8 | AR | SPG62 | ERLIN1 | AR |
| ATXIA Syndrome | ERCC6 | AR | SPG63 | KIF1C | AR |
| ATXIA Syndrome | Aprataxin | AR | SPG64 | USP8 | AR |
| ATXIA Syndrome | ANO10 | AR | SPG65 | WDR48 | AR |
| ATXIA Syndrome | Twinkle | AR | SPG66 | AMPD2 | AR |
| ATXIA Syndrome | PLA2G6 | AR | SPG67 | ENTPD1 | AR |
| ATXIA Syndrome | DARS2 | AR | SPG68 | NT5C2 | AR |
| ATXIA Syndrome | CLCN2 | AR | SPG69 | ARSI | AR |
| ATXIA Syndrome | SIL1 | AR | SPG70 | PGAP1 | AR |
| ATXIA Syndrome | POLG | AR | SPG71 | FLRT1 | AR |
| ATXIA Syndrome | SLC17A5 | AR | SPG72 | RAB3GAP2 | AR |
| ATXIA Syndrome | KCNJ10 | AR | SPG73 | MARS | AR |
| ATXIA Syndrome | POLR3A | AR | SPG74 | ZFR | AR |
| ATXIA Syndrome | PRPS1 | XR | SPG7 | REEP2 | AD/AR |
| ATXIA Syndrome | ATP2B3 | XR | SPG | MAG | AR |
| ATXIA Syndrome | SLC9A6 | XR | SPG | BICD2 | AR |
| ATXIA Syndrome | MECP2 | XR | SPG | LYST | AR/AD |
| ATXIA Syndrome | FMR1 | XR | SPG+HSN | FAM134B | AR |
| Paroxysmal ataxia I | KCNA1 | AD | SPG+HSN | CCT5 | AD |
| Paroxysmal ataxia II | CACNA1A | AD | Paroxysmal ataxia VI | SLC1A3 | AD |
| Paroxysmal ataxia V | CACNB4 | AD | Paroxysmal ataxia VII | SLC2A1 | AD |

| Gene | Inheritance patterns | Gene | Inheritance patterns | Gene | Inheritance patterns | Gene | Inheritance patterns |
| --- | --- | --- | --- | --- | --- | --- | --- |
| ETFDH | AR | GAD1 | AR | TTPA | AR | CASK | AR |
| MARS2 | AR | ARX | AR | C10orf2 | AR | CUL4B | XR |
| ABCD1 | XR | ATRX | AR | CSTB | AR | DKC1 | AR |
| PARK2 | AR | AAAS | AR | EIF2B2 | AR | INPP5E | AR |
| TH | AR | CYP27A1 | AR | EIF2B3 | AR | OPHN1 | AR |
| GCH1 | AR | SLC25A15 | AR | EIF2B5 | AR | PRNP | AD |
| MUT | AR | GALC | AR | FOXC1 | AR | SOD1 | AD |
| MMACHC | AR | IDUA | AR | HSD17B4 | AR | HEXA | AR |
| GCDH | AR | DARS | AR | PMM2 | AR | KANK1 | AR |
| MTHFR | AR | GJA1 | AR | RNF216 | AR | IFIH1 | AR |
| ARG1 | AR | RNF170 | AR | SEPSECS | AR | MRE11A | AR |
| ARSA | AR | SLC30A10 | AR | SETX | AD | TENM3 | AR |
| CBS | AR | NEU1 | AR | TSEN2 | AR | PHYH | AR |
| IDS | XR | ALDH3A2 | AR | TSEN34 | AR | TFG | AD |
| PCCA | AR | ALG6 | AR | TSEN54 | AR | LMNB1 | AD |
| PCCB | AR | ALS2 | AR | VPS53 | AR | MTTP | AR |
| ETHE1 | AR | APTX | AR | ABCB7 | XR | NBN | AR |
| EXOSC3 | AR | ATP7B | AR | C10orf12 | AR | ATP6AP2 | XR |

AD: Autosomal dominant inherited mode; AR: Autosomal recessive inherited mode
